# Molecular Insights into Aqueous NaCl Electrolytes Confined within Vertically-oriented Graphenes

**DOI:** 10.1038/srep14652

**Published:** 2015-10-01

**Authors:** Zheng Bo, Huachao Yang, Shuo Zhang, Jinyuan Yang, Jianhua Yan, Kefa Cen

**Affiliations:** 1 State Key Laboratory of Clean Energy Utilization, Institute for Thermal Power Engineering, College of Energy Engineering, Zhejiang University, Hangzhou, Zhejiang Province, 310027, China

## Abstract

Vertically-oriented graphenes (VGs) are promising active materials for electric double layer capacitors (EDLCs) due to their unique morphological and structural features. This study, for the first time, reports the molecular dynamics (MD) simulations on aqueous NaCl electrolytes confined within VG channels with different surface charge densities and channel widths. Simulation results show that the accessibility of ions and the structure of EDLCs are determined by the ion type/size, surface charging, and VG channel width. For relatively narrow VG channels with the same width, the threshold charge density (to compensate the energy penalty for shedding hydration shell) and the dehydration rate of Cl^−^ ions are larger than those of Na^+^ ions. To achieve the highest ion concentration coefficient, the effective VG channel width should be between the crystal and hydration diameters of the ions. The results are further quantified and elucidated by calculating the electrolyte density profiles. The molecular insights obtained in the current work are useful in guiding the design and fabrication of VGs for advancing their EDLC applications.

Vertically-oriented graphenes (VGs), stacks of graphene nanosheets arranged perpendicularly to a substrate surface, are very attractive for a wide range of energy and environmental applications^1^,^2^. Particularly, VGs have been demonstrated as promising active materials for electric double layer capacitors (EDLCs) due to their unique morphological and structural features^3^,^4^,^5^. For example, the open intersheet channels of VGs can facilitate the ion migration between graphene layers, making the alternating current line-filtering (120 Hz) possible^6^; the vertical orientation of VGs can enhance the charge transport within active materials, leading to outstanding power performance and excellent rate capabilities^7^; the non-agglomerated morphology of VGs with exposed edge planes can promote the charge storage and increase the electrochemically-accessible surface area^6^,^8^. While it has been revealed that the capacitive behaviors of VGs can be tailored by tuning their morphology and structure^9^, an insightful atomistic understanding on the structure of VG-based EDLCs is quite essential in guiding the material optimization.

Molecular dynamics (MD) has been recognized as a powerful technique to describe the motion of particles at molecular scale. It was first accomplished by Alder and Wainwright in 1957 for a condensed phase system^10^. Until now, MD has been widely applied for the EDLC systems employing various carbon-based electrodes, such as planar graphenes^11^,^12^,^13^,^14^, carbon nanotubes (CNTs)^15^,^16^,^17^, onion-like carbons^18^, and activated carbons^19^. The influences of pore geometry/size^15^,^19^, electrolyte chemical structure^11^,^13^,^14^,^16^, temperature^12^,^13^,^14^,^17^, and applied voltage^12^,^17^ on EDLC structure and capacitive behaviors have been extensively investigated. For planar graphene electrodes, the structure of EDLCs was unveiled with MD simulation, providing the mechanisms on the pack of ions within planar pores and its influence on the capacitance^11^,^12^,^13^. For electrodes with curved morphologies such as CNTs and onion-like carbons, the plot of differential capacitance versus potential presented a flat shape^17^,^18^, which is obviously different with those of planar electrodes (bell or camel shape)^12^,^13^; meanwhile, the capacitance increased with a decreasing curvature^18^. Moreover, for electrolytes confined within CNT nanopores, the specific capacitance normalized to the pore surface area was influenced by the pore size in a nonmonotonic manner^15^. Recently, MD simulation was applied to a coconut shell activated carbon with complex shape and irregularly connected pore structure^19^. However, to the best of our knowledge, such an atomistic level simulation on VG-based EDLCs has not been reported.

In this work, MD was employed to examine the structure and charge distribution of aqueous NaCl electrolytes confined within VG channels. The influences of charge density and channel width on the ion/molecule distributions were investigated in detail. The packing behavior ions inside VG channels with different widths and surface charge densities were studied. Furthermore, the EDLC structures within VG channels were quantified by analyzing the electrolyte density profiles and the fundamental interfacial electrolyte properties. The as-obtained molecular insights into the aqueous electrolytes confined within VG channels will be instructive in designing the morphology and structure of VGs for high performance EDLCs.

## Results

**Figure 1:**
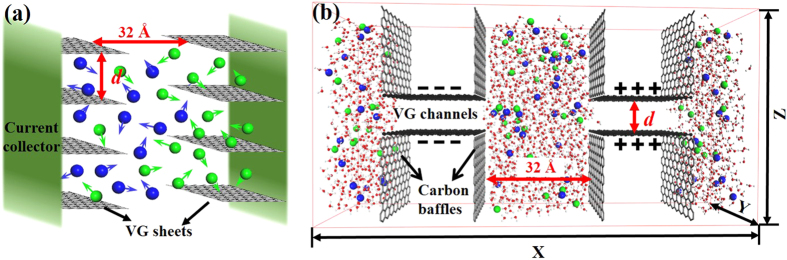
Models of VG-based EDLCs. (**a**) Schematic illustration of a VG-based EDLC. (**b**) Representative snapshot of MD system. Black spheres: carbon atoms with uniform charges; gray sticks: non-charged carbon baffles; red spheres: oxygen atoms in water molecules; white spheres: hydrogen atoms in water molecules; blue spheres: Na^+^ ions; green spheres: Cl^−^ ions.

Figure 1a shows the schematic illustration of a typical VG-based EDLC consisting of a pair of VG electrodes, electrolytes, and two current collectors. The VG growth direction is perpendicular to the current collector, consistent with the direction of ion transport. VG channels are formed by the adjacent graphene sheets. As shown in Fig. 1b, the corresponding MD model was then built with connecting two opposite VG electrodes to a 1.8 M NaCl electrolyte bath. The dimensions of the simulation box were set as 108 and 21.3 Å in X and Y directions, respectively. The electrolyte bath was enclosed by eight carbon baffles, which were not charged during the simulation. The separation between two VG electrodes was 32 Å, where the electrolyte can maintain bulk-like behavior in the middle region of the bath. The dimensions of an individual VG sheet were set as L_x _= 22.1 Å and L_y _= 21.3 Å, respectively. Both values are long enough to eliminate the edge effects from the entrance.

The VG channel widths were set as 6.5, 7, 7.9, 12, and 16 Å, respectively. This width range was paid particular attention, since abnormal EDLC behaviors were reported for the pores with width approaching the crystal and hydration diameters of ions^15^,^20^,^21^,^22^,^23^,^24^. Chialvo *et al.* reported that water might not fill the neutral pore with width less than 6.5 Å^21^. Feng *et al.* revealed that the capacitance of the micropore increased anomalously at the pore width of 7 Å^22^. Kalluri *et al.* found that maximum partition coefficient for Cl^−^ ions was at pore width of *d *= 7.9 Å^23^. On the other hand, the surface charge densities on VG channels were set as 0, ±2, ±4, ±5, ±10, and ±15 μC cm^−2^, falling in the range of −20 μC cm^−2^ to 30 μC cm^−2^ for typical EDLCs employing aqueous electrolytes^25^. These surface charge densities were also investigated by Kalluri *et al.* for EDLC system employing aqueous electrolytes and carbon-slits^23^.

**Figure 2:**
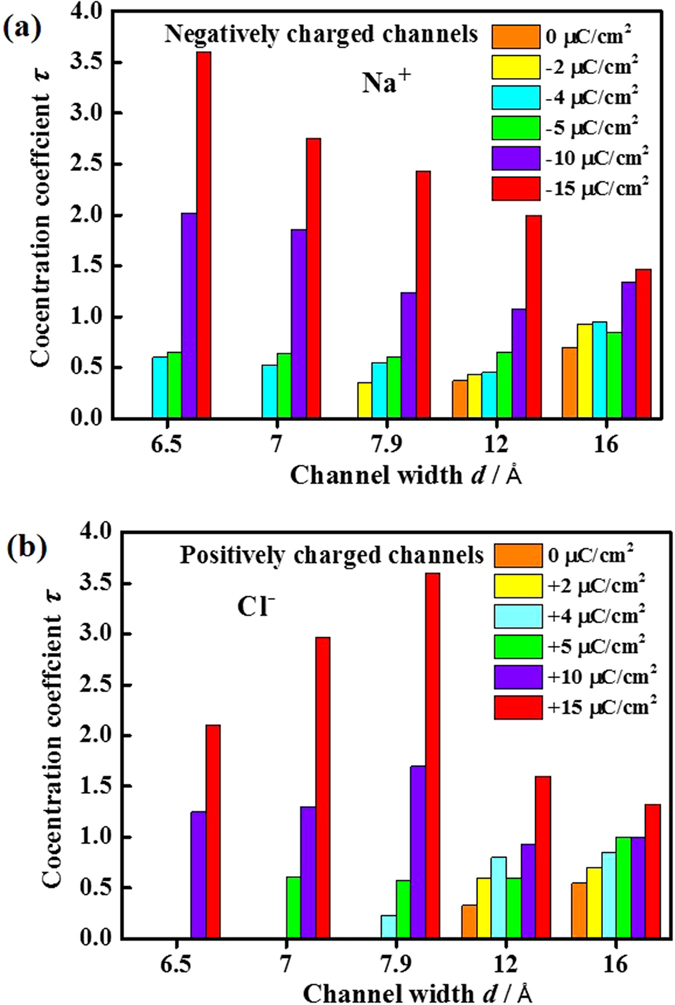
Concentration coefficients of ions. The concentration coefficient *τ* for (**a**) Na^+^ and (**b**) Cl^−^ ions as a function of channel widths of *d *= 6.5, 7, 7.9, 12, and 16 Å at surface charge densities of 0, ±2, ±4, ±5, ±10, and ±15 μC cm^−2^.

It has been extensively demonstrated that the energy storage behaviors of EDLCs are strongly related to the amount of ions accumulated on the electrode^14^,^15^,^23^,^26^,^27^,^28^. To describe the ion packing behavior, a series of coefficients were proposed, *e.g.*, the packing factor^28^, the partition coefficient^23^, the location of ions^28^, and the differential capacitance^14^,^15^. Based on which, two coefficients (*r* and *τ*) were introduced herein to describe the concentration and position of electrolytes confined by VG channels, where *r* is the distance between the first layer of electrolytes and VG charged surface, and the concentration coefficient *τ* is defined as:
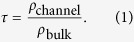


ρ_channel_ and ρ_bulk_ represent the concentrations of electrolytes inside VG channels and in the bulk region, respectively. Figure 2 shows the as-calculated *τ* values of Na^+^ and Cl^−^ ions within the counter-charged VG channels as a function of channel width.

In the case of without charging on VG (*i.e.*, surface charge density = 0 μC cm^−2^), Na^+^ ions can only permeate into the channels of *d *= 12 and 16 Å (*τ*  = 0.37 and 0.7, respectively). No Na^+^ ion was observed within the neutral VG channels of *d* = 6.5, 7, and 7.9 Å (*τ* = 0). This phenomenon can be explained by the relationship between the hydration diameter of ions and the effective VG channel width. The hydration diameter *d*_ion-h_ of Na^+^ ions is 6.6 Å^29^. The effective VG channel width *d′* can be calculated as:
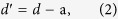
where a is the Lennard-Jones (LJ) diameter of a carbon atom (~ 3.35 Å). For *d* = 6.5, 7, 7.9, 12, and 16 Å, *d′* are calculated as 3.15, 3.65, 4.55, 8.65, and 12.65 Å, respectively. As a consequence, Na^+^ ions can only enter the relatively wide neutral channels, *i.e.*, *d′ *> 6.6 Å. Furthermore, for VG channels of *d *= 12 and 16 Å, *τ* are less than unity (0.37 and 0.7, respectively), suggesting that ions are energy unfavorable inside the channels due to the geometrical confinement^26^. Similar results were also observed for Cl^−^ ions, as shown in Fig. 2b. According to the current simulation, Cl^−^ ions can only enter the neutral VG channels of *d *= 12 and 16 Å, where *d′* is large than the hydration diameter *d*_ion-h_ of Cl^−^ (~7.2 Å)^29^.

The increase of surface charge density will facilitate the accessibility of ions in the relatively narrow VG channels (*i.e.*, *d′ *< *d*_ion-h_), which could be attributed to the desolvation phenomenon^15^,^20^,^24^,^30^. For example, for Na^+^ ions in the channel of *d *= 6.5 Å, *τ* was calculated as 0 and 0.6 at the surface charge densities of 2 and 4 μC cm^−2^, respectively. It suggests that once the surface charge density reaches a threshold, the attractive electrostatic interactions can compensate the energy penalty of stripping part of the hydration shell of ions. An obvious increase of the area specific capacitance in sub-nanometer pores was previously revealed based on experimental observation^26^. According to the current simulation, the threshold charge densities of Na^+^ ions for *d *= 6.5, 7, and 7.9 Å were in the range of 2 ~ 4, 2 ~ 4, and 0 ~ 2 μC cm^−2^, respectively. The threshold charge density of Cl^−^ ions (5 ~ 10, 4 ~ 5, and 2 ~ 4 μC cm^−2^ for *d *= 6.5, 7, and 7.9 Å, respectively) is larger than that of Na^+^ ions at the same channel width. In general, the threshold charge density increased with the decreasing channel width and the increasing hydration diameter of ions. It is worth noting that the setting and the position of the graphene layers can influence the simulation results. Kalluri *et al.* previously performed MD simulation on aqueous electrolyte within carbon-slits^23^. Five graphene layers were used to separate two oppositely charged carbon-slits. The threshold for Na^+^ ions at *d *= 6.5 Å was reported as 1 ~ 2 μC cm^−2^, lower than the result in the current work (2 ~ 4 μC cm^−2^). This result could be attributed to the reduced entrance energy penalty by dispersive interactions between the ions and channel surface, induced by the addition of carbon layers^31^. From this point of view, the current simulation model consisting of two opposite graphene electrodes fits the real VG morphology well, providing a better understanding on its energy storage behaviors.

**Figure 3:**
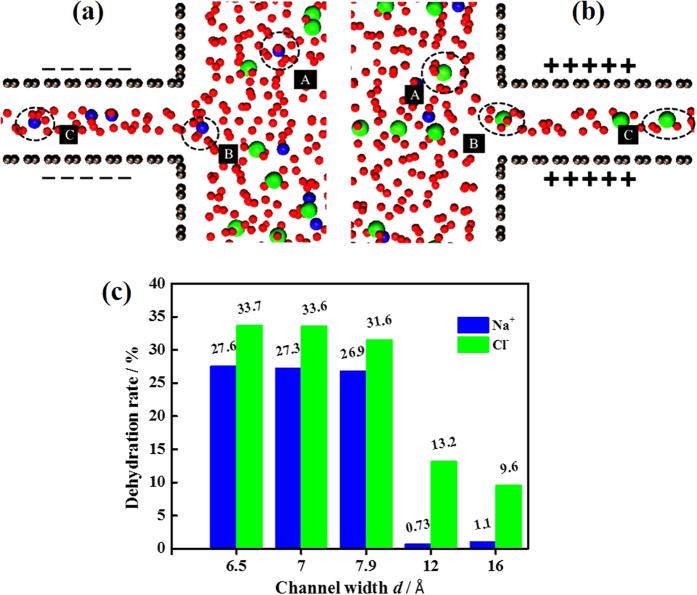
Dehydration of ions within charged VG channels (surface charge density = ±15 μC cm^−2^). Representative simulation snapshots of (**a**) Na^+^ and (**b**) Cl^−^ ions within VG channels (*d *= 7 Å) at the positions of A (in bulk region), B (at VG channel entrance), and C (inside VG channel). (**c**) Dehydration rates of Na^+^ and Cl^−^ ions within the VG channels of *d *= 6.5, 7, 7.9, 12, and 16 Å.

As is well known, the hydration diameter of Cl^−^ ions (*d*_ion-h _= 7.2 Å) is larger than that of Na^+^ ions (*d*_ion-h _= 6.6 Å). As a consequence, the hydration shell of Cl^−^ ions is more distorted with permeating into narrow VG channels, as shown in Fig. 3a,b, and thus a higher threshold charge density is needed. It is consistent with previous reports that the energy barrier of Cl^−^ ion is higher than that of Na^+^ ion when entering the narrow pores^31^,^32^. To quantify the distortion of hydration shell within VG channels, the dehydration rate, defined as the percentage for stripping the hydration water molecules of ions, was calculated. Figure 3c shows the dehydration rates of Na^+^ and Cl^−^ ions as a function of VG channel width. The hydration number for Na^+^ and Cl^−^ ions in the bulk region were calculated as 5.5 and 7.6, respectively, consistent with previously reported values^31^. As shown in Fig. 3c, for the same VG channel width, the dehydration rate of Cl^−^ was larger than that of Na^+^ ions.

**Figure 4:**
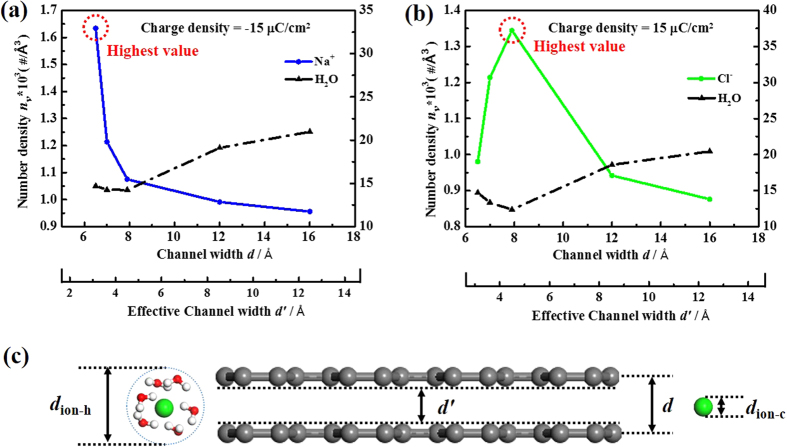
Relationship between VG channel width and number densities within channels (surface charge density = ±15 μC cm^−2^). Number densities (*n*_*v*_) of (**a**) Na^+^ ions/water molecules and (**b**) Cl^−^ ions/water molecules at various VG channel widths. The left and right Y axes present ions (solid lines) and molecules (dashed lines), respectively. (**c**) Schematic illustration of the optimized VG channel width.

Once the surface charge density is above the threshold, the highest *τ* value was achieved at a certain VG channel width. As shown in Fig. 2, the optimum VG channel widths for Na^+^ and Cl^−^ ions were 6.5 Å and 7.9 Å, respectively. To elucidate the observed results, number density *n*_*v*_ is introduced to describe the occupancy of ions and molecules inside the VG channels, defined as:
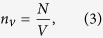


where *N* presents the number of ions or molecules within VG channels, and *V* presents the volume of VG channels. Figure 4a,b show the number densities (*n*_*v*_) of ions and water molecules as a function of channel width at a relatively high surface charge density of ±15 μC cm^−2^. For wide VG channels (*d′ *> *d*_ion-h_, *e.g.*, *d *= 12 Å and 16 Å), the ions can accumulate with relatively low dehydration rates (see Fig. 3c), residing at a position slightly further from the channel surface. It could result in lower ion number density and higher water number density (*e.g.*, *d *= 16 Å for Na^+^, as shown in Fig. 4a), further leading to relatively low *τ* values. On the other hand, as the VG channel width approaches the crystal diameter of ions (*d*_ion-c_), more parts of hydration shell were shed (see Fig. 3c), which significantly decreased the water densities (*e.g.*, *d *= 7.9 Å for Cl^−^, as shown in Fig. 4b) and led to a higher *τ* values. The distance between ions and channel surface (*r*) was also reduced (Supplementary Information, Fig. S1). However, a further decrease of the VG channel width could lead to the decrease of *τ* value (*e.g.*, *d *= 7.0 Å and 6.5 Å for Cl^−^, as shown in Fig. 2b). Previous experimental observation suggests that either larger or smaller pores can lead to a significant drop of capacitance^30^. The above results indicate that the highest *τ* value can be obtained with setting the effective VG channel width between the crystal and hydration diameters of the ions (*i.e.*, *d*_ion-c _< *d′ *< *d*_ion-h_, as shown in Fig. 4c). This result agrees well with the experimental observations, where a 100% increase in normalized capacitance was observed as the channel/pore size approached the crystallographic diameter of ion^20^. Our MD simulation results suggest that the energy storage behaviors of VG-based EDLCs can be optimized by precisely controlling the channel widths with the consideration of ion size.

## Discussion

To better understand the microstructure of EDLCs inside VG channels, the effects of charge density and channel width were elucidated by examining the electrolyte density profiles. The area-based number densities of ions and molecules at the position of *z* along the direction perpendicular to VG nanosheets, *i.e.*, *n*(*z*), is defined as:
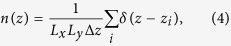


where Σ_*i*_δ(*z–z*_*i*_) gives the number of water molecules and ions inside a layer of thickness *Δz*.

The area number density profiles in a narrow channel (*d *= 7.9 Å, *d′ *< *d*_ion-h_) and a wide channel (*d *= 12 Å, *d′ *> *d*_ion-h_) were compared in detail. More data for the channel widths of *d *= 6.5, 7, and 16 Å can be found in Supplementary Information, Fig. S2.

**Figure 5:**
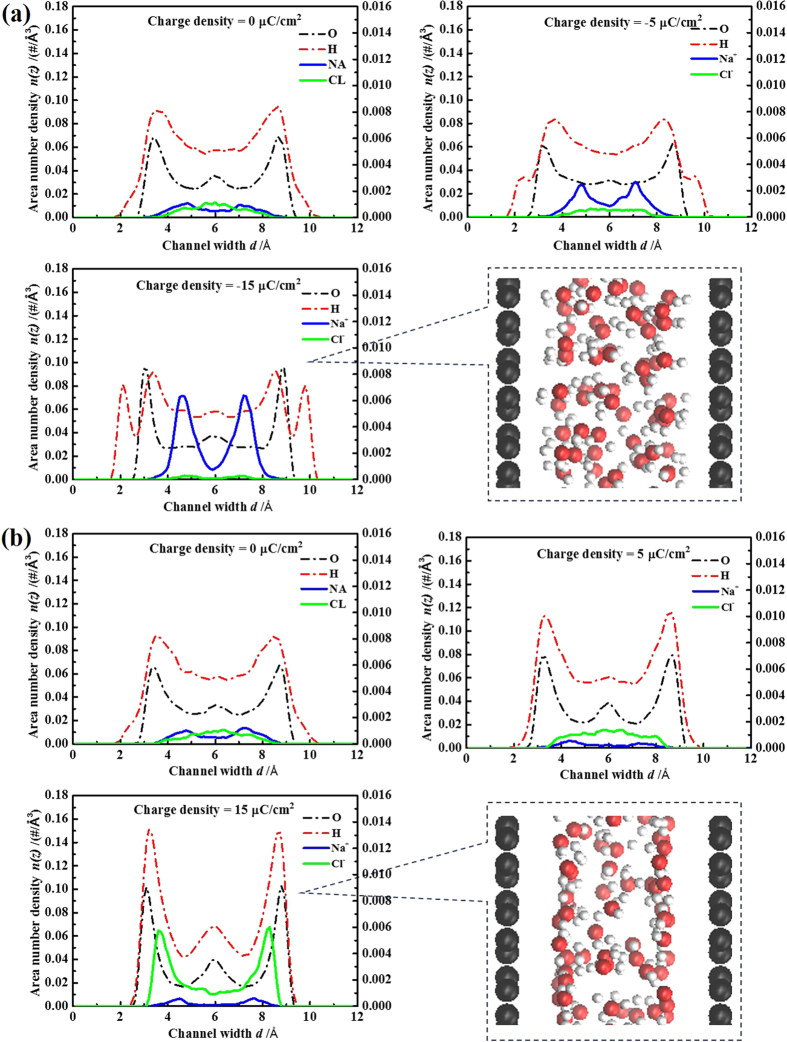
Distribution of electrolytes in VG channel of *d *= 12 Å. Area number density profiles of ions and water molecules within (**a**) negatively and (**b**) positively charged channels of *d *= 12 Å. The left Y axes present the density of atomic oxygen (black dashed lines) and hydrogen (red dashed lines) in water molecules. The right Y axes present the densities of Na^+^ ions (blue solid lines) and Cl^−^ ions (green solid lines).

Figure 5 shows the area number density profiles of ions and molecules within the VG channel of *d *= 12 Å. With an increasing charge density, the number of water molecules within VG channels varied a little (Supplementary Information, Fig. S3), but more molecules were adsorbed to the channel surface, presenting a multiple distinct layer structure. For the same absolute value of charge density, the orientation of water molecules within negatively and positively charged VG channels was found to be very different. This phenomenon was obvious especially at a relatively high surface charge density of ±15 μC cm^−2^, as shown in Fig. 5. In positively charged VG channels, the O and H peaks were almost at the same z position and the water molecules were probably in the plane parallel to the VG channel surface. In negatively charged VG channels, the O peak was sandwiched by two H peaks, which could be attributed to the channel-water electrostatic interactions (one of the H atoms could be attracted by the negatively charged carbon atoms). The distribution of Na^+^ and Cl^−^ ions within VG channels also varied with an increasing surface charge density. Without charging, a small number of Na^+^ and Cl^−^ ions were observed in the center of VG channels, consistent with the aforementioned results on ion concentration coefficient (see Fig. 2). With the increase of surface charge density, the EDLC structure was achieved by attracting counter-ions into VG channel and expelling the co-ions to the bulk region. The change of ion numbers as a function of the charge density can be found in Supplementary Information, Fig. S4. For the relatively high surface charge density of ±15 μC cm^−2^, obvious EDLC structures were constructed, consisting of two dense layers of ions contacting the charged surface. Cl^−^ ions resided at a position embed inside the first H atoms of water molecules, while Na^+^ ions accumulated at a position depleted of water molecules. It suggests a favorable interaction between Cl^−^ ions and H atoms of water molecules^27^. Meantime, there were a small number of co-ions found in the center of the negatively charged channels, probably due to the ion-ion correlations^33^. For wider channel of *d *= 16 Å, the structure of EDLCs (see Supplementary Information, Fig. S2) was similar to that of *d *= 12 Å.

**Figure 6:**
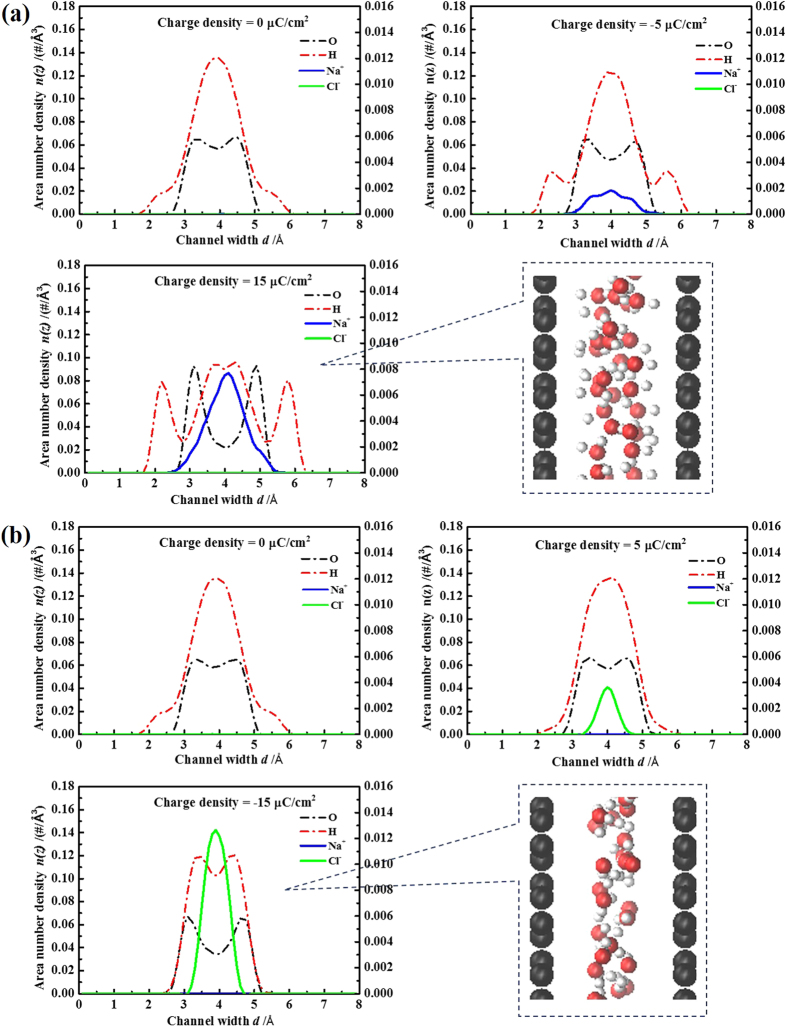
Distribution of electrolytes in VG channel of *d *= 7.9 Å. Area number density profiles of ions and water molecules within (**a**) negatively and (**b**) positively charged channels of *d *= 7.9 Å. The left Y axes present the density of atomic oxygen (black dashed lines) and hydrogen (red dashed lines) in water molecules. The right Y axes present the densities of Na^+^ ions (blue solid lines) and Cl^−^ ions (green solid lines).

The structures of EDLCs within narrow VG channels are obviously different with the wide channel counterparts. Figure 6 shows the area number density profiles of ions and molecules within the VG channel of *d *= 7.9 Å. The water layers in the channel with a surface charge density of ±15 μC cm^−2^ presented an overlapped structure due to the geometrical confinement. No ion was observed in neutral channels. With an increasing surface charge density, the structure of EDLCs was achieved by inserting the counter-ions without expelling the co-ions, showing a different charging behavior for wide VG channels (see Fig. 5). Besides, the ion-ion correlations are absent in narrow channels. The structure of EDLCs within *d *= 7.9 Å channel mainly consisted of a single layer of ions. Na^+^ ions developed a broader layer than that of Cl^−^ ions, especially for surface charge density of ±15 μC cm^−2^, which is mainly due to size effects. Similar results were observed for narrower VG channels *d *= 7 and 6.5 Å (see Supplementary Information, Fig. S2).

**Figure 7:**
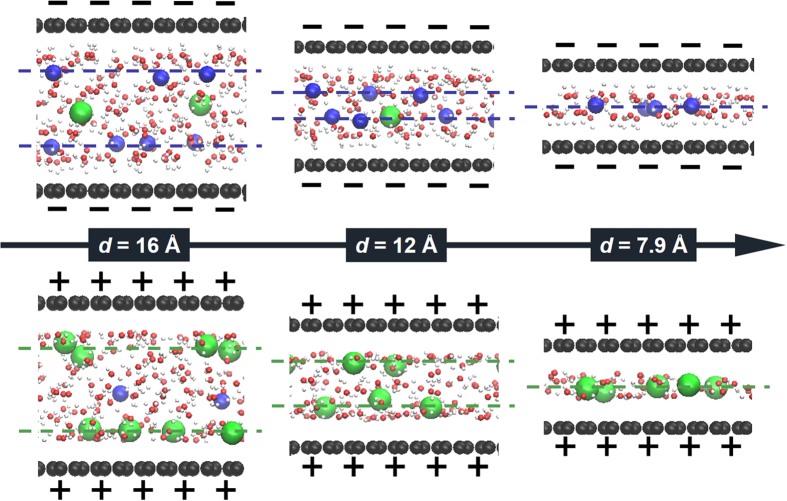
Simulation snapshots of electrolytes within VG channels. Representative simulation snapshots of ions and molecules at the surface charge densities of ±15 μC cm^−2^ within VG channels of *d *= 7.9, 12, and 16 Å. Blue and green dashed lines denote the layers of Na^+^ and of Cl^−^ ions, respectively.

The representative simulation snapshots of ions and molecules inside VG channels are displayed to visualize the effects of channel width on the structure of electrolytes. Figure 7 shows the simulation snapshots of ions and molecules on the surface charge density of ±15 μC cm^−2^ within channel widths of *d *= 7.9, 12, and 16 Å. Data for other two widths, i.e., *d *= 7.0 and 6.5 Å are presented in Supplementary Information, Fig. S5. For negatively charged VG channels, with a decreasing width from 16 to 12 Å, the distance between two Na^+^ layers decreased from 6.7 to 2.2 Å. With a further decrease of the channel width to 7.9 Å, a single chain of electrolytes was observed due to the geometrical confinement. Similar observation was also obtained for positively charged VG channels. These results are consistent with the area number density profiles shown in Figs 5 and 6.

## Conclusions

In summary, MD simulation was performed on the NaCl electrolytes confined within VG channels, with focusing on the influences of channel width and surface charge density on the structure of EDLCs and charge distribution. For the cases without surface charging, ions can only enter the relatively wide VG channels (*d′ *> *d*_ion-h_), and an external surface charging is needed to conquer the energy barrier encountered by the ions as they enter narrow VG channels (*d′ *< *d*_ion-h_). For the same VG channel width, the threshold charge density and the dehydration rate of Cl^−^ ions are larger than those of Na^+^ ions. To achieve the highest *τ* values, the effective VG channel width is suggested to be at the range between the crystal and hydration diameters of the ions. The above results are corroborated by the electrolyte density profiles, which present totally different charging behaviors and EDLC structures within narrow and wide VG channels. The formation of EDLCs in wide VG channels relies on the exchange of counter-ions and co-ions, while only the inserting of counter-ions occurs for the narrow counterparts. The geometrical confinement in narrow VG channels will also significantly influence the electrode-electrolyte interfacial behaviors and the ion/molecule distributions. The results obtained in the current atomistic MD simulations provide useful insight for advancing the optimization of VG-based supercapacitors. In addition to the current simulation system (aqueous electrolytes within VG channels), similar approach can be applied to more complex cases (*e.g.*, hierarchical structures such as CNTs-on-VG^34^ and VG-on-CNTs^35^, non-aqueous electrolytes, and hybrid energy storage devices containing redox reactions). Proper accounting of the electronic polarizability and flexibility of electrodes will be worthwhile in our future work for a more rigorously quantitative analysis.

## Methods

To fit the real situation, all carbon atoms in the current MD model were kept rigid and fixed during the current simulation. The intra-molecular or bonded terms of carbon atoms in VGs, including bond stretching, angle bending, and dihedral torsions, were neglected. Carbon atoms in VGs were modeled as LJ spheres with the potential parameters from Cheng and Steele^36^, which were widely used to capture the interfacial behaviors in graphene systems with fixed carbon atoms^37^,^38^. The water molecules were characterized by the simple point charge extended model (SPC/E)^39^,^40^,^41^,^42^. The constraint algorithm was applied for the stretching terms between oxygen and hydrogen atoms to reduce high frequency vibrations^43^. The Na^+^ and Cl^−^ ions were modeled as charged LJ particles with the parameters proposed by Dang^44^. In these models, the interactions between atomic sites (or ions) are expressed as a sum of Columbic and LJ 12-6 potentials:
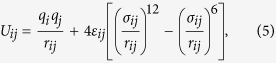


where *q*_*i*_, *r*_*ij*_, *ε*_*i*_, and *σ*_*i*_ represent the charge of *i*th atom or ion, the distance between *i*th and *j*th atom or ion, the minimum energy and the zero energy separation distance, respectively. The LJ parameters for *ε*_*ij*_ and *σ*_*ij*_ are obtained by using the Lorentz-Berthelot mixing rules^45^.

The MD simulations were performed in the canonical ensemble with the Large-scale Atomic/Molecular Massively Parallel Simulator (LAMMPS) program^46^. The results were visualized by VMD software. Periodic boundary condition was applied for all the three directions. All simulations were simulated in the NVT ensemble at the temperature of 300 K. The temperature was controlled by a Nose-Hoover thermostat with a 100 fs damping parameter^47^. A cutoff distance of 9 Å was used for Van der Waals and electrostatic interactions in the real space. The long-range electrostatic interactions were treated by the particle mesh Ewald summation method^48^ with a root mean-square accuracy of 10^−4^. The velocity Verlet algorithm were employed to integrate Newton’s equation of motion with a time step of 1 fs^49^. Each system was relaxed for 5 ns, and the simulations were run for another 5 ns to obtain the average quantities discussed.

## Additional Information

**How to cite this article**: Bo, Z. *et al.* Molecular Insights into Aqueous NaCl Electrolytes Confined within Vertically-oriented Graphenes. *Sci. Rep.* **5**, 14652; doi: 10.1038/srep14652 (2015).

## Supplementary Material

Supplementary Information
