# Supplementary material for: Molecular Insights into Aqueous NaCl Electrolytes Confined within Vertically-oriented Graphenes
Source: Sci Rep. 2015 Oct 1;5:14652. doi: 10.1038/srep14652 (PMC4589769; doi:10.1038/srep14652)
Supplement: Supplementary Information [file srep14652-s1.doc]

Supplementary Information

Molecular Insights into Aqueous NaCl Electrolytes Confined within Vertically-oriented Graphenes

Zheng Bo*, Huachao Yang, Shuo Zhang, Jinyuan Yang, Jianhua Yan & Kefa Cen

State Key Laboratory of Clean Energy Utilization, Institute for Thermal Power Engineering, College of Energy Engineering, Zhejiang University, Hangzhou, Zhejiang Province, 310027, China

Correspondence should be addressed to Zheng Bo (Email: bozh@zju.edu.cn; Tel: 86 571 87953290)

**1. Distance between the ions and charged VG channel surface.**

**Figure S1.** Distance *r* between the first layer of ions and charged VG channel surface as a function of channel width *d* = 6.5, 7, 7.9, 12, and 16 Å.

**2. Distribution of electrolytes in VG channels.**

**
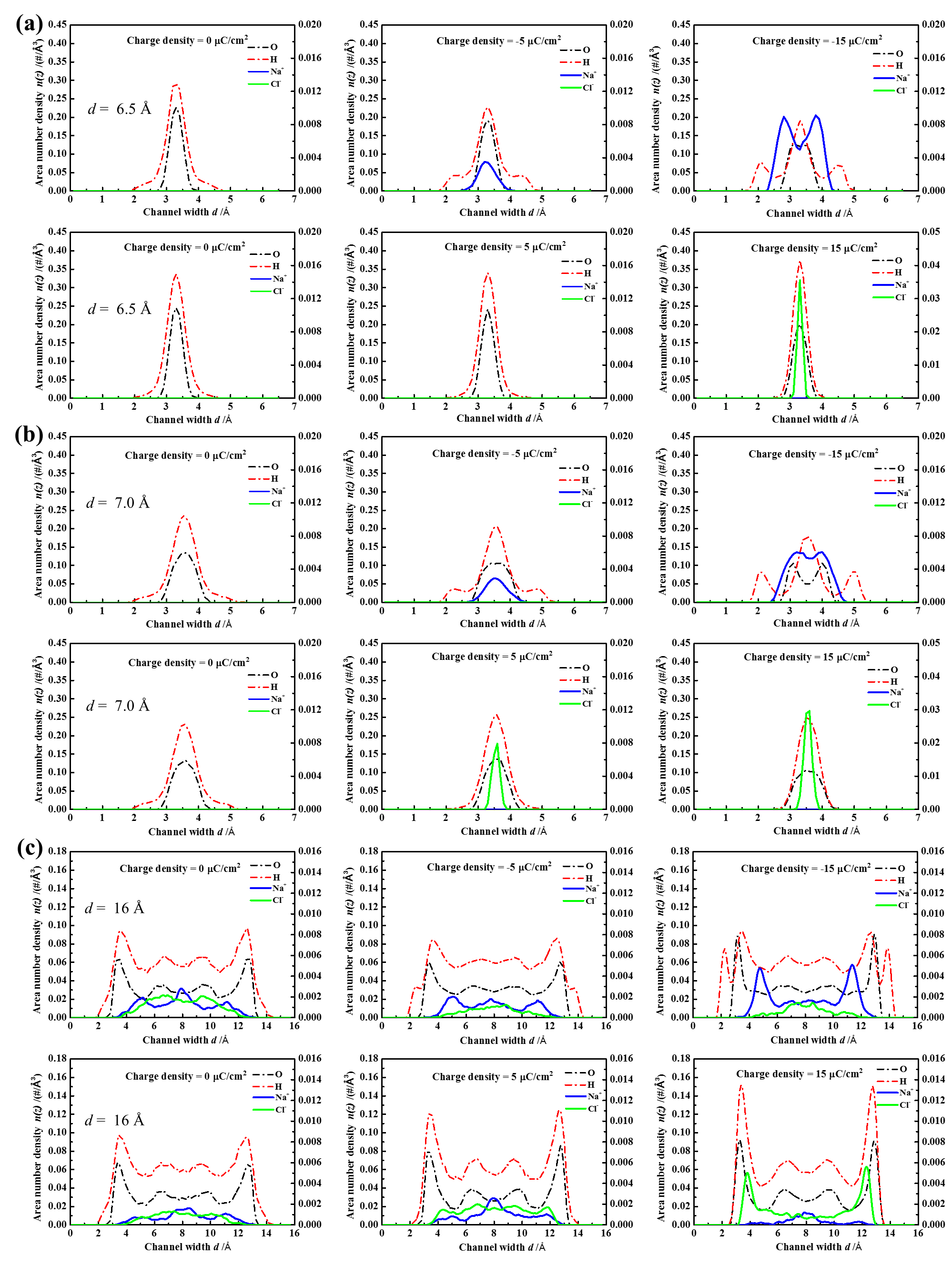
**

**Figure S2. Distribution of electrolytes in VG channel of 6.5, 7, and 16 Å.** Area number density profiles of ions and water molecules within VG channels of (a) *d* = 6.5 Å, (b) *d* = 7.0 Å, and (c) *d* = 16 Å as a function of surface charge density 0, ±5, and ±15 μC cm^-2^. The left Y axes present the area number densities of atomic oxygen (black dashed lines) and hydrogen (red dashed lines) in water molecules. The right Y axes present the area number densities of Na^+^ (blue solid lines) and Cl^-^ (green solid lines).

**3.** **Number of water molecules as a function of surface charge density.**


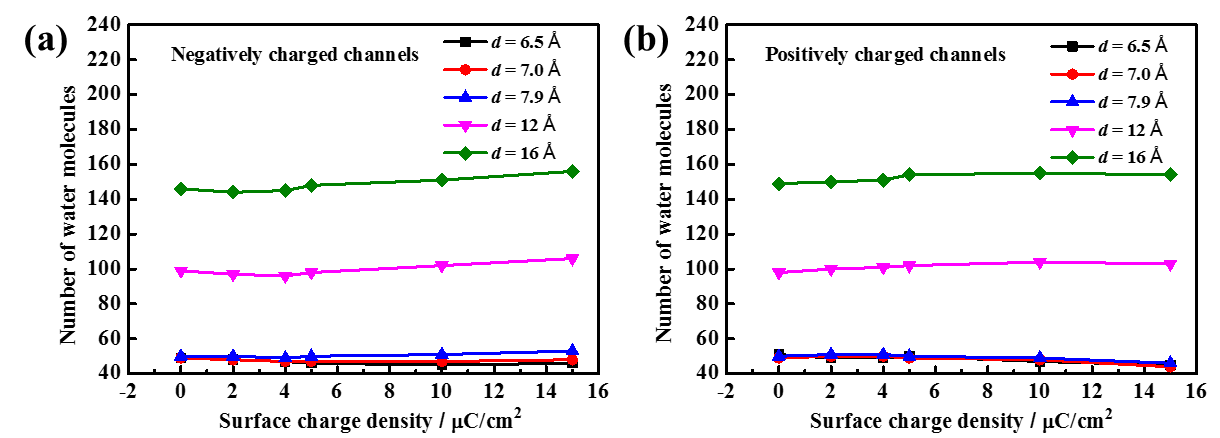


**Figure S3. Number of water molecules in VG channels.** Average number of water molecules at surface charge densities of 0, ±2, ±4, ±5, ±10, and ±15 μC cm^-2^ within (a) negatively and (b) positively charged VG channels.

**4. Number density (*n_v_*) of ions as a function of surface charge density.**

**
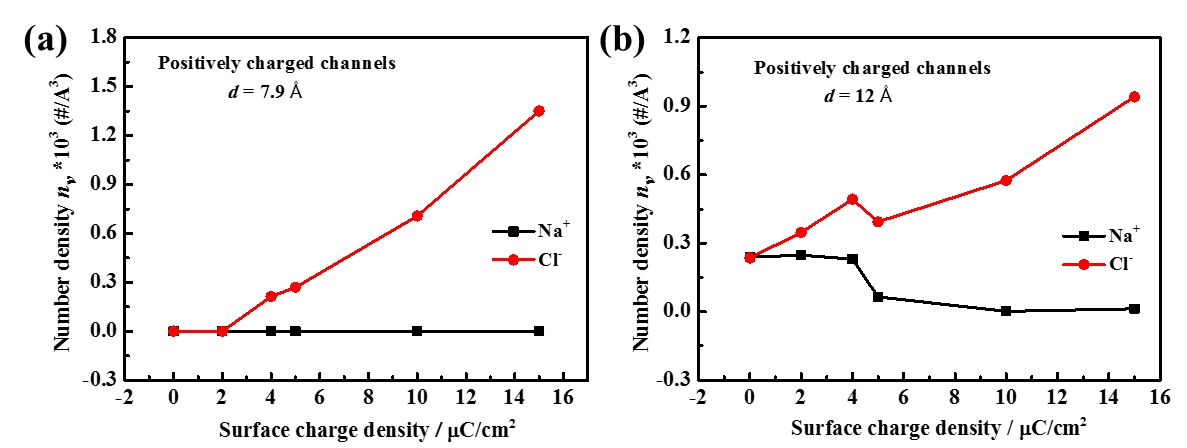
**

**Figure S4. Number density (*n_v_*) of ions as a function of surface charge density.** Number density (*n_v_*) of ions at surface charge densities of 0, 2, 4, 5, 10, and 15 μC cm^-2^ within (a) *d* = 7.9 Å and (b) *d* = 12 Å VG channels.

**5. Representative simulation snapshots of electrolytes within VG channels.**

**
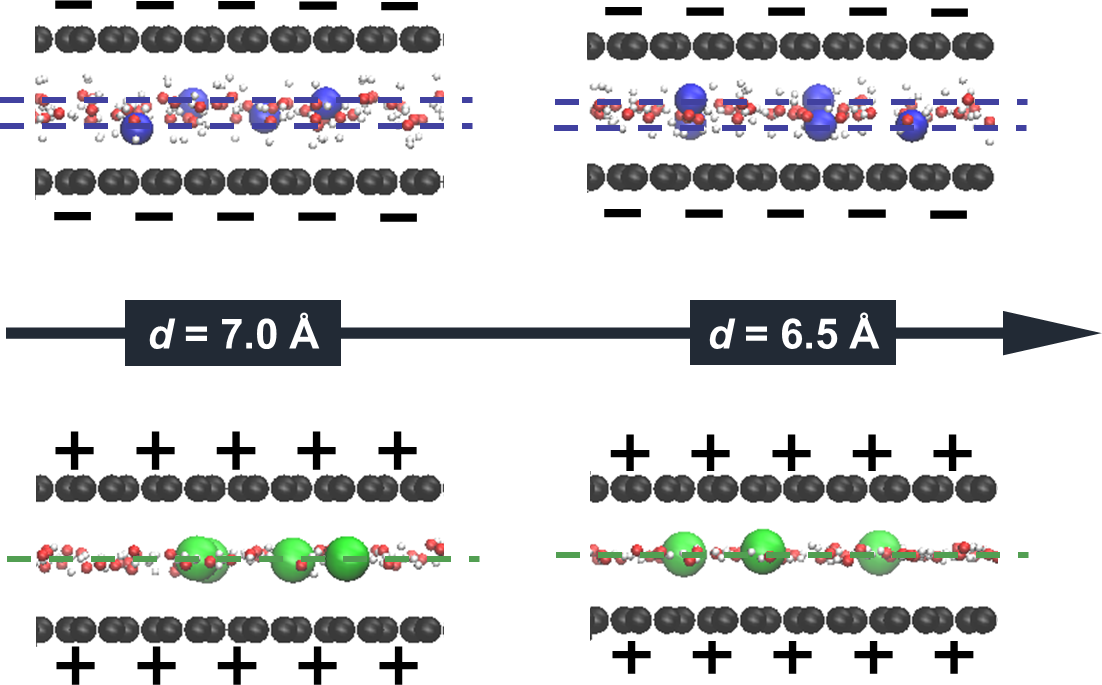
**

**Figure S5. Simulation snapshots of electrolytes within VG channels.** Representative simulation snapshots of ions and molecules at the surface charge densities of ±15 μC cm^-2^ within VG channels of *d* = 7.0 and 6.5 Å. Blue and green dashed lines denote the layers of Na^+^ and of Cl^-^ ions, respectively.
